# Integrative Analysis of Vitamin D–Associated Genetic Variants Reveals Cis‐Regulatory Architecture and Multigenic Mechanisms Underlying Chronic Disease–Relevant Pathways

**DOI:** 10.1155/humu/3304798

**Published:** 2026-05-13

**Authors:** Jia Yue, Jinqing Zhang, Ke Xu, Ping Han, Bing Tan

**Affiliations:** ^1^ Department of School of Public Administration, Chongqing Finance and Economics College, Chongqing, China, cqcfe.com; ^2^ Department of Human Resources, Chongqing Electric Power College, Chongqing, China; ^3^ Department of Oncology, Chongqing General Hospital, Chongqing University, Chongqing, China, cqu.edu.cn; ^4^ Department of Pharmacy, Chongqing General Hospital, Chongqing University, Chongqing, China, cqu.edu.cn

**Keywords:** cis-regulatory variants, chronic disease pathways, gene-level aggregation, genome-wide association study, vitamin D genetics

## Abstract

Vitamin D is a pleiotropic regulator of immune, metabolic, and endocrine homeostasis and has been implicated in a broad spectrum of chronic diseases. Although genome‐wide association studies (GWAS) have identified numerous genetic variants associated with vitamin D–related traits, most signals reside in noncoding regions, limiting biological interpretation and disease‐oriented translation. Here, we present an integrative analytical framework to interpret vitamin D–associated genetic variation by linking variant‐level associations to cis‐regulatory architecture, gene‐level aggregation, and functional organization. Vitamin D–related traits were curated from the GWAS Catalog using ontology‐guided criteria. Genome‐wide significant variants were functionally annotated and mapped to nearby genes using a standardized ± 50‐kb cis‐regulatory window. Independent variants were aggregated at the gene level to prioritize robust candidate genes for downstream analysis. We found that vitamin D–associated variants were widely distributed across the genome and were predominantly enriched in noncoding regulatory regions. Cis‐regulatory mapping revealed extensive SNP–gene multiplicity, reflecting complex local regulatory architectures. Gene‐level aggregation identified a prioritized set of genes supported by multiple independent variants, which converged on pathways central to chronic disease biology, including immune regulation, metabolic processes, endocrine signaling, and intracellular signal transduction. Network‐based integration further revealed modular regulatory structures characterized by coordinated pathway convergence. Together, these results indicate that vitamin D–associated genetic variation contributes to chronic disease susceptibility through coordinated cis‐regulatory and multigenic mechanisms rather than isolated gene effects.

## 1. Introduction

Vitamin D plays a central role in maintaining physiological homeostasis across multiple organ systems, with well‐established functions in bone metabolism, immune modulation, and endocrine regulation [1, 2, 3]. In addition to its classical role in calcium–phosphate metabolism, increasing epidemiological and experimental evidence suggests that vitamin D status is associated with a wide range of chronic diseases, particularly metabolic disorders, immune‐mediated conditions, and endocrine‐related pathologies [4]. However, interindividual variability in vitamin D levels and downstream biological responses remain substantial, suggesting an important contribution of genetic determinants to vitamin D–related phenotypes and disease susceptibility.

Genome‐wide association studies (GWAS) have identified numerous genetic variants associated with circulating vitamin D levels and vitamin D–related traits [5, 6, 7]. Notably, the majority of these variants reside in noncoding regions of the genome, complicating direct biological interpretation [8, 9]. While individual loci have been linked to vitamin D synthesis, transport, and metabolism, many genome‐wide significant signals still lack clear functional annotation or confident gene assignment [10]. This disconnect between statistical association and biological mechanism represents a major barrier to translating vitamin D genetic findings into interpretable biomarkers or mechanistic insights relevant to chronic disease.

Emerging evidence indicates that noncoding variants frequently exert their effects through cis‐regulatory mechanisms, influencing gene expression by modulating regulatory elements rather than protein sequence [11]. Such variants may affect multiple genes within a local genomic neighborhood, giving rise to complex, multigenic regulatory architectures [12]. These observations highlight the need for analytical frameworks that extend beyond single‐variant or single‐gene interpretations and instead consider gene‐level integration and network‐level organization.

Gene‐level aggregation approaches provide a principled strategy to integrate multiple independent genetic signals converging on the same gene, thereby enhancing robustness and biological interpretability [13, 14]. By combining gene‐level aggregation with cis‐regulatory mapping, it becomes possible to systematically prioritize genes supported by convergent genetic evidence rather than relying on individual association signals alone. Downstream functional enrichment and network‐based analyses can then place prioritized genes into coherent biological contexts, revealing shared pathways and regulatory modules that may underlie chronic disease–relevant processes [15].

In this study, we developed an integrative analytic framework to systematically interpret vitamin D–associated genetic variation from variant‐level associations to gene‐level aggregation and pathway‐level organization. Specifically, we integrated genome‐wide significant GWAS variants, cis‐regulatory SNP–gene mapping, gene prioritization based on independent variant support, and functional and network analyses to characterize the biological pathways potentially influenced by vitamin D–associated genetic variation. Rather than focusing on individual loci, this framework emphasizes coordinated genetic effects and pathway‐level organization that may help explain how vitamin D–related genetic variation contributes to chronic disease susceptibility.

## 2. Methods

### 2.1. Data Acquisition and Preprocessing

Genome‐wide association data for vitamin D–related traits were retrieved from the GWAS Catalog [16] using ontology‐guided trait selection [17] to ensure relevance to vitamin D metabolism, regulation, and related physiological phenotypes. Traits were selected based on Experimental Factor Ontology (EFO) annotations and keyword matching related to vitamin D biology (e.g., vitamin D levels, 25‐hydroxyvitamin D, and related metabolic traits). For each trait, summary statistics including effect size estimates, standard errors, and association *p* values were extracted. Variants reaching genome‐wide significance (*p* < 5 × 10^−8^) were retained for downstream analyses [18]. Genomic coordinates and reference SNP identifiers were standardized to a common genome build, and duplicated entries were removed.

### 2.2. Genome‐Wide Distribution and Annotation of Significant Variants

Chromosome‐wise distributions of genome‐wide significant SNPs were summarized to assess large‐scale genomic patterns [19]. SNP positions were ordered along each chromosome to generate cumulative genomic density profiles. Functional annotations were obtained from variant consequence fields associated with each SNP [20]. Variants were classified into coding and noncoding categories based on predicted impact on protein‐coding sequences. Coding variants were further subdivided into missense, stop‐gained, frameshift, and splice‐related classes. rsID‐based quality control was performed to distinguish reference SNPs from non‐rsID variants [19]. Variant summarization and visualization were performed in R using tidyverse utilities, and figures were generated with ggplot2.

### 2.3. SNP–Gene Mapping

Genome‐wide significant SNPs were mapped to genes using a cis‐regulatory window defined as ± 50 kb from SNP genomic positions. This distance threshold was selected based on commonly used proximity‐based mapping strategies in GWAS interpretation studies, which aim to capture potential local regulatory effects while limiting excessive gene assignment. Gene coordinates were obtained from the GENCODE gene annotation database (release version specified) [21]. Any gene whose transcription start site or gene body intersected the cis window was retained as a candidate target for the corresponding SNP. SNP–gene overlaps were determined using interval‐based genomic intersection operations implemented in R [22]. For each SNP, the number of mapped genes was recorded to quantify regulatory multiplicity.

### 2.4. Gene‐Level SNP Aggregation and Candidate Gene Prioritization

Mapped SNPs were aggregated at the gene level to derive gene‐specific SNP burdens. Independent SNPs were defined as distinct genome‐wide significant variants reported in the GWAS Catalog, and no additional LD‐based pruning was performed because only lead variants from published associations were included. For each gene, the total number of independent genome‐wide significant SNPs within the cis window was counted. Candidate gene sets were defined under increasing SNP‐burden thresholds (≥ 1, ≥ 2, and ≥ 3 SNPs). The number of retained genes at each threshold was summarized to evaluate trade‐offs between robustness and coverage. Genes supported by at least two independent SNPs were selected as the final prioritized candidate set for downstream analyses.

### 2.5. Functional Enrichment Analysis

Prioritized candidate genes were subjected to gene ontology biological process and KEGG pathway enrichment analyses using over‐representation testing [23, 24]. Enrichment analyses were conducted in R using established functional annotation and enrichment packages, including clusterProfiler [25], with default parameters unless otherwise specified. Multiple testing correction was applied to obtain adjusted *p* values. Enrichment results were summarized by selecting significantly enriched terms and pathways for visualization. Functional similarity among enriched GO terms was evaluated to identify clusters of related biological processes [26].

### 2.6. Construction of Gene–Pathway Bipartite Networks

Significantly enriched KEGG pathways (adjusted *p* < 0.05) were retained for network construction. Gene–pathway relationships were defined based on gene membership within each enriched pathway. These relationships were represented as bipartite networks consisting of gene nodes and pathway nodes connected by undirected edges. Network construction and analysis were performed using standard R network analysis frameworks.

### 2.7. Module Definition and Hub Gene Identification

Functional modules were defined by grouping pathways into higher order biological themes, including immune, metabolic, endocrine, and signal transduction/cellular regulation. Module assignment was based on KEGG functional classification and manual curation according to biological similarity. Genes were assigned to modules based on their connected pathways. Within each module, hub genes were identified using degree centrality, defined as the number of pathways connected to each gene.

### 2.8. Network Visualization and Statistical Analysis

Gene–pathway networks were visualized using force‐directed layouts. Node type (gene or pathway), functional category, and connectivity were encoded using node color, size, and spatial arrangement to enhance interpretability. Network construction and visualization were performed in R using standard network analysis and visualization packages.

### 2.9. Statistical Analysis

All statistical analyses were conducted in R. Descriptive statistics were used to summarize variant counts, gene counts, SNP burden distributions, and enrichment results. Multiple testing correction for enrichment analyses was performed using adjusted *p* values. Unless otherwise specified, analyses were performed using standard parameters implemented in the corresponding R packages.

## 3. Results

### 3.1. Overview of the Analytical Framework

To systematically dissect the genetic architecture underlying vitamin D–related traits, we established an integrative analytical framework that links genome‐wide association signals to gene‐level functional interpretation (Figure S1). Vitamin D–associated traits were curated from the GWAS Catalog through ontology‐guided selection, and corresponding association statistics were subsequently retrieved. Genome‐wide significant variants (*p* < 5 × 10^−8^) were then annotated with genomic coordinates and functional consequence information. To facilitate biological interpretation, significant SNPs were mapped to nearby genes using a ± 50‐kb cis‐regulatory window. Genes supported by multiple independent variants were prioritized as candidate targets, enabling downstream functional interpretation through gene ontology and KEGG pathway enrichment analyses.

### 3.2. Genome‐Wide Landscape of Vitamin D–Associated Genetic Variation

**Figure 1:**
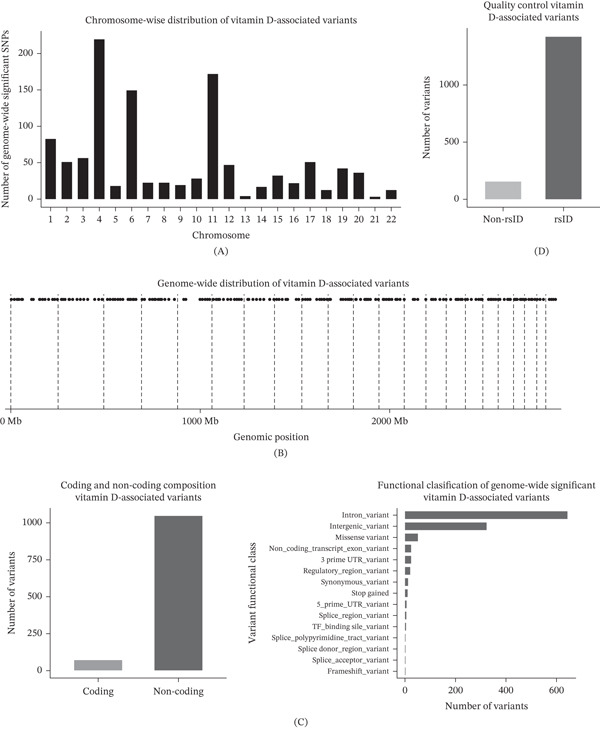
Genome‐wide landscape of vitamin D–associated variants. (A) Chromosome‐wise distribution of genome‐wide significant SNPs associated with vitamin D–related traits, (B) cumulative genomic density of significant SNPs ordered by chromosomal position highlighting regional clustering patterns, (C) proportion of variants located in coding versus non‐coding genomic regions with functional subclassification of coding variants, and (D) quality control assessment of variant annotation comparing rsID‐annotated SNPs with non‐rsID variants retained in the analysis.

To establish a global view of genetic variation associated with vitamin D–related traits, we analyzed genome‐wide significant variants identified from curated GWAS datasets. Chromosome‐wise aggregation revealed that significant SNPs were distributed across all autosomes rather than concentrated at a limited number of loci (Figure 1A). Cumulative genomic density analysis further revealed uneven clustering patterns along chromosomal coordinates, suggesting regional enrichment of association signals rather than uniform genomic distribution (Figure 1B). Functional annotation showed that the majority of variants were located in noncoding genomic regions, with only a small fraction residing within protein‐coding sequences (Figure 1C). Among coding variants, missense substitutions constituted the predominant class, whereas predicted loss‐of‐function events such as stop‐gained and frameshift variants were comparatively rare. Quality control based on rsID annotation indicated that most retained variants corresponded to well‐characterized reference SNPs, supporting the robustness of downstream analyses (Figure 1D).

### 3.3. Functional Architecture of Protein‐Altering and Regulatory Variants

**Figure 2:**
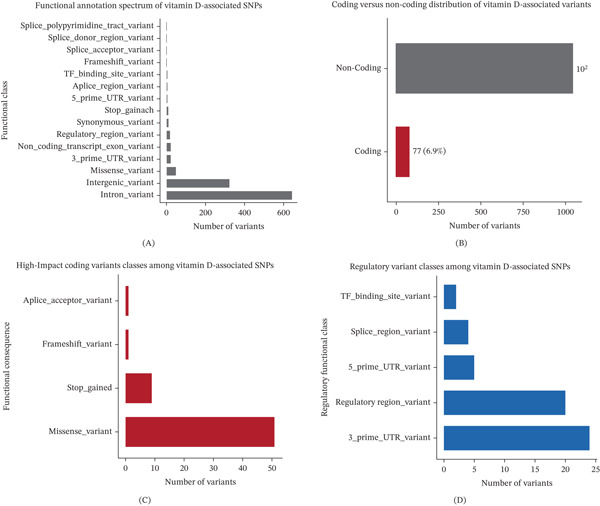
Functional annotation landscape of vitamin D–associated variants. Distribution of (A) functional consequence categories among genome‐wide significant SNPs associated with vitamin D–related traits, (B) proportion of variants located in coding versus noncoding genomic regions, (C) functional subclassification of protein‐impactful variants including missense, stop‐gained, frameshift, and splice‐related annotations, and (D) distribution of regulatory‐associated variant classes including untranslated regions, regulatory regions, splice regions, and transcription factor binding site–related annotations.

We next examined the functional composition of genome‐wide significant variants in greater detail. Protein‐altering variants represented a minor subset of the overall association signals, with missense variants accounting for the largest proportion, followed by splice‐related and truncating events (Figure 2A). In contrast, variants with regulatory annotations, including untranslated regions, splice regions, and regulatory element–associated variants, accounted for the majority of the variant landscape (Figure 2B). Quantitative comparison of functional classes highlighted a marked imbalance between coding and regulatory variation, underscoring the predominance of noncoding mechanisms among vitamin D–associated loci (Figure 2C). These findings suggest that most vitamin D–associated loci may exert their effects through regulatory mechanisms rather than through direct alteration of protein sequences (Figure 2D).

### 3.4. Cis‐Regulatory SNP–Gene Mapping Reveals Extensive Regulatory Multiplicity

**Figure 3:**
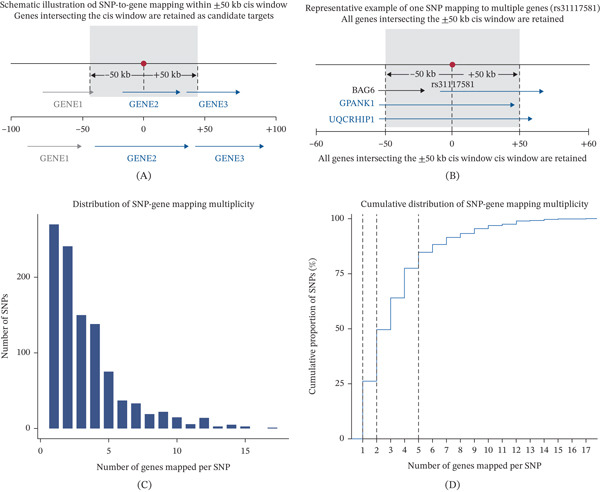
SNP–gene mapping and candidate gene prioritization. (A) Schematic illustration of the SNP‐to‐gene mapping strategy using a ± 50‐kb cis‐regulatory window in which all genes intersecting the window are retained as candidate targets, (B) distribution of SNP–gene mapping multiplicity showing the number of genes mapped per SNP across all genome‐wide significant variants, (C) cumulative distribution of SNP–gene mapping multiplicity illustrating the proportion of SNPs captured as a function of the number of mapped genes, and (D) workflow for candidate gene prioritization based on SNP support highlighting the selection of genes mapped by at least two independent SNPs for downstream analyses.

To link associated variants to potential target genes, we mapped genome‐wide significant SNPs to nearby genes using a ± 50‐kb cis‐regulatory window. This analysis showed that a substantial proportion of SNPs were linked to more than one gene, indicating widespread regulatory multiplicity at vitamin D–associated loci (Figure 3A). The distribution of SNP–gene mapping counts showed a long‐tailed pattern, with most variants linked to one or two genes but a non‐negligible subset mapping to larger gene sets (Figure 3B). Cumulative mapping analysis further demonstrated that multigene assignments represented a common pattern rather than isolated cases (Figure 3C). Representative loci illustrated that single regulatory variants can simultaneously intersect multiple gene bodies or promoters within the cis window (Figure 3D), highlighting the complexity of local regulatory architecture underlying vitamin D genetic associations.

### 3.5. Gene‐Level Aggregation Identifies Robust Candidate Targets

**Figure 4:**
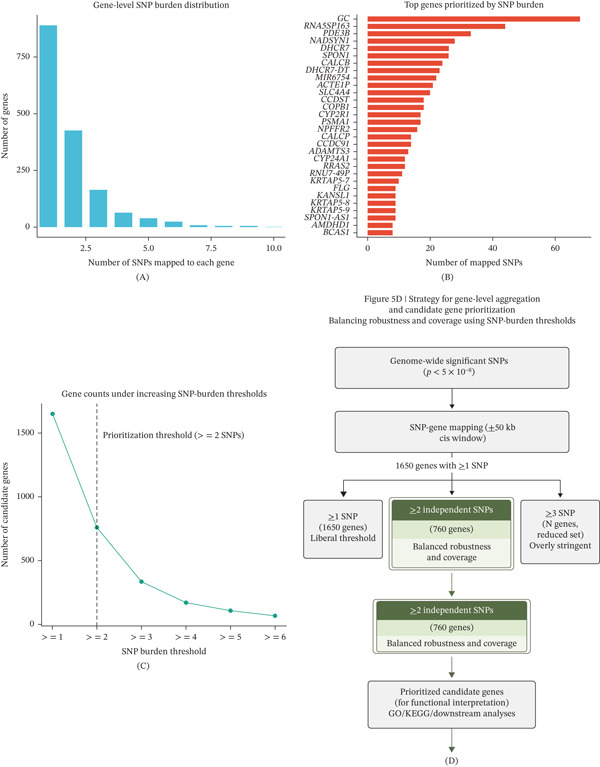
Gene‐level aggregation of genome‐wide significant vitamin D–associated variants and prioritization strategy. (A) Distribution of SNP burden across all genes mapped within a ± 50‐kb cis window illustrating heterogeneity in gene‐level variant accumulation, (B) top‐ranked genes prioritized by SNP burden highlighting genes with disproportionately high numbers of mapped SNPs, (C) changes in the number of candidate genes retained under increasing SNP‐burden thresholds demonstrating the trade‐off between stringency and gene coverage, and (D) schematic overview of the gene‐level aggregation and prioritization strategy illustrating selection of genes with ≥ 2 independent SNPs as a balanced criterion for robustness and downstream functional interpretation.

To consolidate variant‐level signals into gene‐level candidates, we aggregated SNPs mapped to each gene and quantified gene‐level SNP burden. A total of 1650 genes harbored at least one genome‐wide significant SNP within the defined cis window. Increasing the SNP‐burden threshold progressively reduced the number of retained genes, with genes supported by two or more independent SNPs representing a balance between robustness and coverage (Figure 4A–C). Genes exceeding more stringent thresholds (≥ 3 SNPs) represented a markedly smaller subset, reflecting increased stringency but reduced gene coverage. Based on this distribution, genes supported by at least two independent SNPs were selected as the prioritized candidate set for downstream functional analyses (Figure 4D).

### 3.6. Functional Convergence of Prioritized Genes Across Biological Pathways

**Figure 5:**
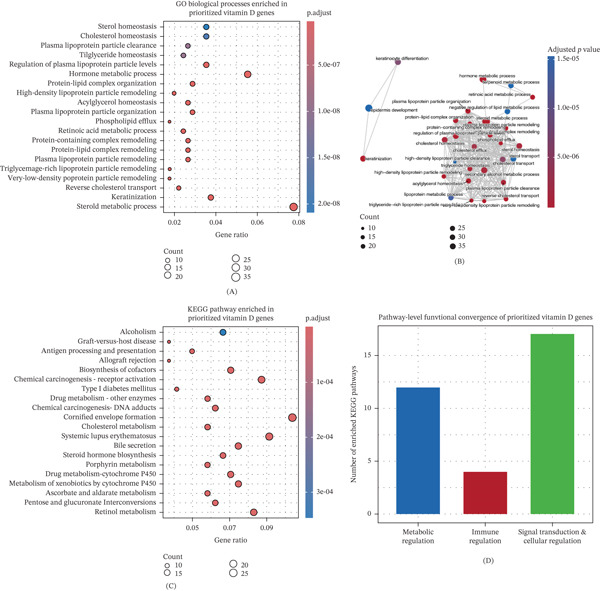
Gene ontology and KEGG functional enrichment analysis of prioritized vitamin D–associated genes. (A) Gene ontology biological process enrichment displayed as a dot plot highlighting the top enriched terms, (B) functional similarity–based clustering of enriched GO biological processes illustrating higher order biological themes, (C) KEGG pathway enrichment analysis of prioritized genes showing significantly overrepresented pathways, and (D) pathway‐level functional summarization of enriched KEGG terms grouping pathways into metabolic regulation, immune regulation, and signal transduction and cellular regulation categories.

Functional enrichment analysis of prioritized candidate genes revealed significant over‐representation of pathways involved in metabolic regulation, immune processes, and signal transduction (Figure 5). Gene ontology biological process enrichment highlighted clusters related to cellular metabolism, immune activation, and regulatory signaling cascades (Figure 5A). KEGG pathway analysis further identified enrichment in pathways associated with xenobiotic metabolism, hormone biosynthesis, immune signaling, and intracellular regulatory networks (Figure 5B). Functional similarity analysis indicated that enriched terms formed biologically coherent clusters rather than representing unrelated processes (Figure 5C). Collectively, these results indicate that vitamin D–associated genes converge on a limited number of interconnected functional programs rather than being dispersed across unrelated pathways (Figure 5D).

### 3.7. Network‐Level Integration Reveals Modular Regulatory Structure

**Figure 6:**
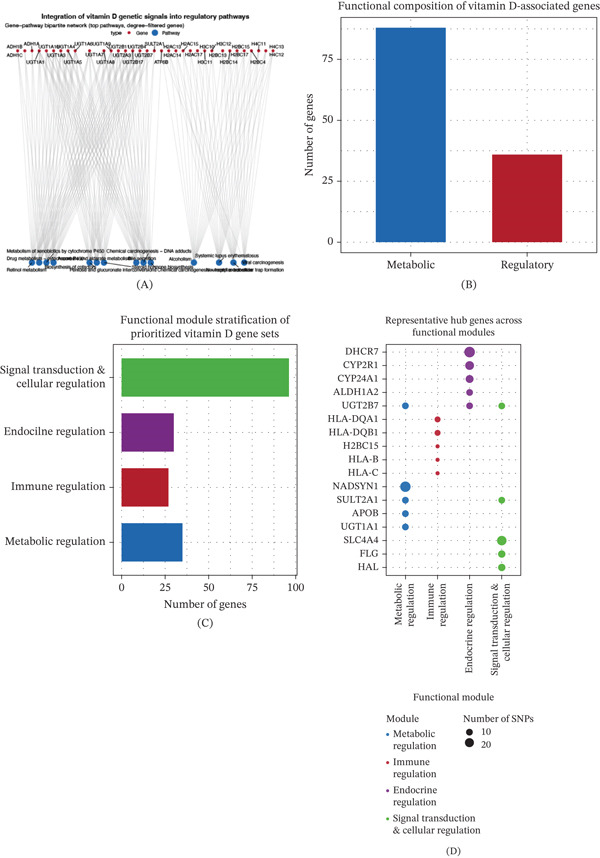
Integration of vitamin D genetic signals into regulatory networks. (A) Gene–pathway bipartite network connecting prioritized vitamin D–associated genes with significantly enriched KEGG pathways illustrating structured gene–pathway relationships, (B) proportional comparison of regulatory/signaling‐related genes versus metabolic genes within the prioritized gene set, (C) network‐based classification of genes into immune, endocrine, metabolic, and signal transduction/cellular regulation modules based on pathway connectivity, and (D) representative hub genes within each functional module defined by high pathway connectivity in the integrated network.

To examine how prioritized vitamin D–associated genes collectively organize beyond individual pathways, we integrated gene–pathway relationships into a bipartite regulatory network. The resulting network revealed a nonrandom architecture in which subsets of genes converged on a limited number of highly connected pathways, forming structured gene–pathway clusters rather than diffuse associations (Figure 6A). Multiple genes were simultaneously connected to pathways related to metabolic detoxification, steroid biosynthesis, immune regulation, and signal transduction, suggesting coordinated pathway‐level organization of vitamin D genetic signals. Quantitative stratification of the prioritized gene set showed that genes involved in regulatory and signaling processes constituted a substantial fraction of the network, comparable to or exceeding the contribution of classical metabolic genes (Figure 6B). Based on pathway connectivity patterns, genes segregated into higher order functional modules corresponding to immune, endocrine, metabolic, and signal transduction–centered axes (Figure 6C). These modules showed partial overlap rather than strict separation, indicating shared genes connecting multiple biological systems. Within each module, a subset of genes displayed disproportionately high pathway connectivity, emerging as module‐specific hubs (Figure 6D). Collectively, these results demonstrate that vitamin D–associated genetic signals integrate into a coordinated regulatory network characterized by pathway convergence, functional modularity, and hub gene organization.

## 4. Discussion

In this study, we present an integrative framework to interpret vitamin D–associated genetic variation with an explicit focus on chronic disease–relevant regulatory organization. Rather than emphasizing individual loci or single‐gene associations, our analyses systematically connect variant‐level signals to cis‐regulatory structure, gene‐level aggregation, pathway convergence, and modular network architecture. This multiscale view suggests that vitamin D genetics may be better interpreted as coordinated regulatory patterns rather than as isolated genetic effects.

A defining feature of the vitamin D genetic landscape uncovered here is the overwhelming predominance of noncoding variation [6, 7]. While this pattern has been observed across many complex traits, its implications are particularly salient for vitamin D, a hormone‐like regulator with pleiotropic effects across immune, metabolic, and endocrine systems [27, 28, 29]. The limited number of protein‐altering variants suggests that genetic influences on vitamin D–related phenotypes are more likely to involve regulatory processes affecting gene expression or pathway activity rather than direct disruption of protein‐coding sequences [11, 30]. Consequently, interpretation strategies that focus narrowly on coding consequences are likely to miss the dominant sources of biological signal.

Our cis‐regulatory mapping analyses further demonstrate that vitamin D–associated variants frequently intersect multiple genes within local genomic neighborhoods [12, 31]. This regulatory multiplicity is not an analytical artifact but a structural property of the genomic regions harboring vitamin D associations. Such complexity challenges simplistic one‐variant–one‐gene interpretations and underscores the need for gene‐level integration strategies. By aggregating variant signals at the gene level, we reduce the impact of local mapping ambiguity and highlight genes supported by convergent genetic evidence [32, 33]. Importantly, the resulting gene set does not expand indiscriminately but instead reflects a constrained and interpretable collection of targets, indicating that regulatory complexity at the variant level resolves into structured organization at the gene level.

Functional analyses of prioritized genes reveal a clear pattern of convergence rather than dispersion [34]. Enriched pathways cluster around biological processes central to chronic disease biology, including immune regulation, metabolic control, endocrine signaling, and intracellular signal transduction. This pattern of convergence is notable because it emerges from data‐driven integration of noncoding variants without requiring prior assumptions about disease mechanisms. The alignment between genetic convergence and known physiological domains of vitamin D action suggests that regulatory variation contributes to chronic disease susceptibility by modulating interconnected systems rather than isolated molecular pathways.

Network‐based integration provides an additional layer of insight by revealing how prioritized genes are organized across pathways [35]. The observed modular structure indicates that vitamin D–associated genes participate in coordinated functional communities, with certain genes occupying central positions that link multiple biological domains. These hub genes may represent potential points of regulatory integration where genetic variation could influence multiple biological processes [36]. From a translational perspective, this network organization offers a principled framework for prioritization: genes that connect multiple pathways or modules may serve as more informative candidates for downstream validation than genes identified solely through enrichment statistics [37].

Building on these analyses, we propose an integrative conceptual framework in which vitamin D–associated genetic variation is primarily organized through cis‐regulatory mechanisms and multigenic functional networks relevant to chronic disease biology. Rather than implying direct causal relationships or disease specificity, this framework provides a structured perspective for interpreting genetic associations within biologically meaningful contexts. By distinguishing variant‐level associations from regulatory organization and pathway‐level convergence, this approach also highlights areas where additional evidence, such as tissue‐specific regulatory data or disease‐oriented functional validation, may further refine biological interpretation.

Several limitations should be considered when interpreting these findings. The use of a fixed cis‐regulatory window provides a consistent and reproducible strategy for SNP–gene mapping but does not account for long‐range chromatin interactions or context‐dependent regulatory effects [38]. Functional annotations and pathway databases reflect current knowledge and may incompletely represent cell‐type–specific regulatory mechanisms relevant to particular chronic diseases [39]. Furthermore, the gene‐level aggregation strategy used here favors robustness by prioritizing genes supported by multiple variants, which may reduce sensitivity to genes influenced by a limited number of strong regulatory signals. Taken together, these limitations indicate that the present framework should be interpreted as a prioritization and organizational strategy rather than as a definitive mechanistic model.

In summary, our study highlights vitamin D genetics as a predominantly regulatory and multigenic landscape in which dispersed noncoding variants converge into coherent functional and network‐level organization. By integrating cis‐regulatory mapping, gene‐level aggregation, functional enrichment, and modular network analysis, we provide a scalable approach for translating vitamin D–associated genetic variation into interpretable biological axes relevant to chronic disease. Future studies incorporating functional validation and disease‐specific datasets will be important to further refine these observations.

## 5. Conclusion

This study provides an integrative framework linking vitamin D–associated genetic variants to cis‐regulatory structure, gene‐level prioritization, and functional networks. Our findings suggest that vitamin D genetics is characterized by coordinated regulatory organization rather than isolated gene effects. These results offer a basis for future studies seeking to interpret vitamin D genetic associations in the context of chronic disease biology.

## Author Contributions

Jia Yue and Ke Xu conceived and designed the study. Jia Yue and Ke Xu conducted the data analysis and generated the visualizations. Jia Yue, Jinqing Zhang, Ke Xu, Ping Han, and Bing Tan contributed to manuscript drafting and critical revision. Ping Han and Bing Tan provided overall supervision and secured funding for the study.

## Funding

No funding was received for this manuscript.

## Disclosure

All authors reviewed and approved the final manuscript.

## Conflicts of Interest

The authors declare no conflicts of interest.

## Supporting information


**Supporting Information** Additional supporting information can be found online in the Supporting Information section. Figure S1: Analytical workflow of the integrative framework used in this study. Schematic overview of the study design illustrating ontology‐guided trait curation, variant annotation, cis‐regulatory SNP–gene mapping, gene‐level aggregation, functional enrichment, and network‐based integration.

## Data Availability

All data analyzed in this study are derived from publicly available resources. Genome‐wide association study (GWAS) summary statistics for vitamin D–related traits were obtained from the GWAS Catalog. All datasets used are publicly accessible, and detailed accession information is provided in the Methods section. The code and intermediate results generated during the current study are available from the corresponding author upon reasonable request.
