# Supplementary figures and images for: Integrative Analysis of Vitamin D–Associated Genetic Variants Reveals Cis‐Regulatory Architecture and Multigenic Mechanisms Underlying Chronic Disease–Relevant Pathways
Source: Hum Mutat. 2026 May 13;2026:3304798. doi: 10.1155/humu/3304798 (PMC13169137; doi:10.1155/humu/3304798)

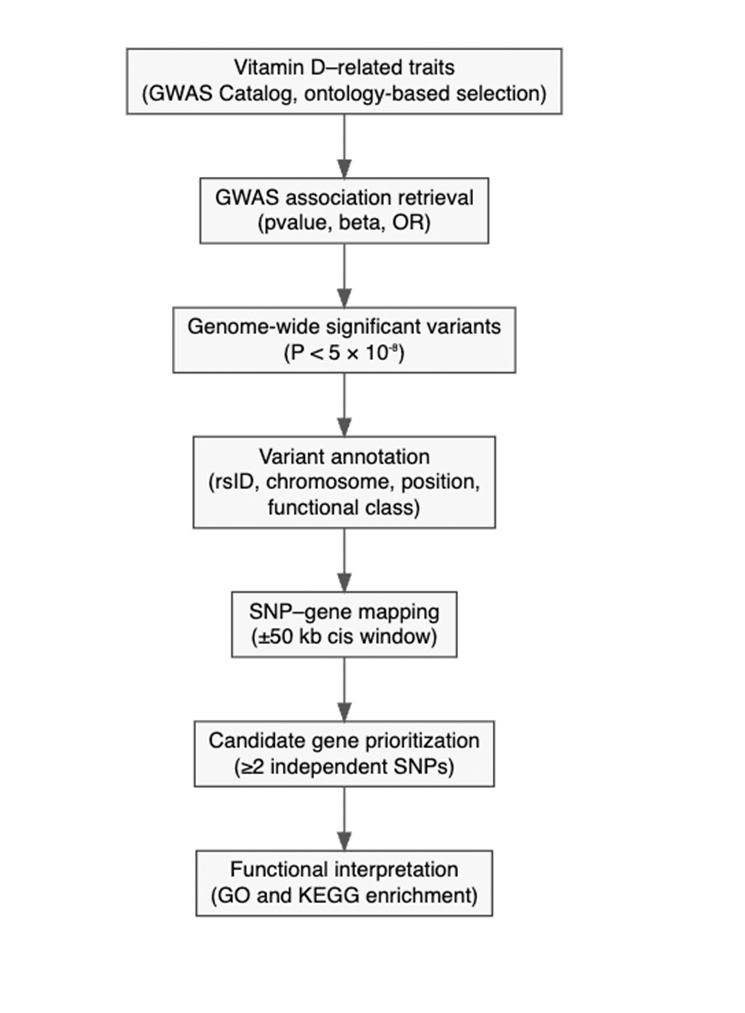

Supplement: Supplementary file 1 — Supporting Information Additional supporting information can be found online in the Supporting Information section. Figure S1: Analytical workflow of the integrative framework used in this study. Schematic overview of the study design illustrating ontology‐guided trait curation, variant annotation, cis‐regulatory SNP–gene mapping, gene‐level aggregation, functional enrichment, and network‐based integration. [file HUMU-2026-3304798-s001.tif]
